# Head-Down Tilt Position, but Not the Duration of Bed Rest Affects Resting State Electrocortical Activity

**DOI:** 10.3389/fphys.2021.638669

**Published:** 2021-02-24

**Authors:** Katharina Brauns, Anika Friedl-Werner, Martina A. Maggioni, Hanns-Christian Gunga, Alexander C. Stahn

**Affiliations:** ^1^Charité – Universitätsmedizin Berlin, a corporate member of Freie Universität Berlin, Humboldt-Universität zu Berlin, and Berlin Institute of Health, Institute of Physiology, Berlin, Germany; ^2^INSERM U 1075 COMETE, Université de Normandie, Caen, France; ^3^Department of Biomedical Sciences for Health, Università degli Studi di Milano, Milan, Italy; ^4^Department of Psychiatry, Perelman School of Medicine, University of Pennsylvania, Philadelphia, PA, United States

**Keywords:** spaceflight, bed rest, brain, EEG, cognition, fluid shift

## Abstract

Adverse cognitive and behavioral conditions and psychiatric disorders are considered a critical and unmitigated risk during future long-duration space missions (LDSM). Monitoring and mitigating crew health and performance risks during these missions will require tools and technologies that allow to reliably assess cognitive performance and mental well-being. Electroencephalography (EEG) has the potential to meet the technical requirements for the non-invasive and objective monitoring of neurobehavioral conditions during LDSM. Weightlessness is associated with fluid and brain shifts, and these effects could potentially challenge the interpretation of resting state EEG recordings. Head-down tilt bed rest (HDBR) provides a unique spaceflight analog to study these effects on Earth. Here, we present data from two long-duration HDBR experiments, which were used to systematically investigate the time course of resting state electrocortical activity during prolonged HDBR. EEG spectral power significantly reduced within the delta, theta, alpha, and beta frequency bands. Likewise, EEG source localization revealed significantly lower activity in a broad range of centroparietal and occipital areas within the alpha and beta frequency domains. These changes were observed shortly after the onset of HDBR, did not change throughout HDBR, and returned to baseline after the cessation of bed rest. EEG resting state functional connectivity was not affected by HDBR. The results provide evidence for a postural effect on resting state brain activity that persists throughout long-duration HDBR, indicating that immobilization and inactivity *per se* do not affect resting state electrocortical activity during HDBR. Our findings raise an important issue on the validity of EEG to identify the time course of changes in brain function during prolonged HBDR, and highlight the importance to maintain a consistent body posture during all testing sessions, including data collections at baseline and recovery.

## Introduction

Future long-duration spaceflight missions (LDSM) will be much longer than current standard missions on the International Space Station (ISS). They will be characterized by increased physiological, environmental, and psychosocial stressors, including, but not limited to weightlessness, hypokinesia, isolation and confinement, radiation, increased CO_2_ levels, and sleep disruptions. Adverse cognitive and behavioral conditions and psychiatric disorders are considered a critical and unmitigated risk during such missions (McPhee and Charles, 2009). Monitoring and mitigating crew health and performance risks during LDSM will require tools and technologies that allow to reliably assess cognitive performance and behavioral health. Functional resting state magnetic resonance imaging (rsfMRI) has considerable potential to predict human behavior. Using rsfMRI, resting state functional connectivity was shown to be associated with a variety of cognitive performance tasks, such as attention (Clare Kelly et al., 2008), working memory (Gordon et al., 2012) and fluid intelligence (Cole et al., 2012), as well as emotional states (Bush et al., 2018; Kim et al., 2019), and stress (Chen et al., 2019; Nowak et al., 2020; Sato et al., 2020). Currently, no MRI system is available on the ISS. Due to size as well as technical and operational requirements, it is rather unlikely that any such system featuring neuroimaging capabilities will be deployed on spacecrafts in the near future. Electroencephalography (EEG) has the potential to meet the technical requirements for the non-invasive and objective monitoring of neurobehavioral conditions during LDSM. Some technologies are readily commercially available that are lightweight, highly mobile, battery-operated, and allow non-invasive recordings of electrical cortical activity (Amaral et al., 2017; Casson, 2019; He et al., 2019). EEG recordings in orbit have been successfully employed as part of the Shuttle mission ‘Neurolab’ (Witten, 2005) and the experiment ‘NeuroSpat’ on the ISS (Cheron et al., 2006, 2014; Cebolla et al., 2016; Petit et al., 2019). Weightlessness induces a considerable fluid shift to the upper body (Thornton et al., 1987). Furthermore, Roberts et al. (2017) reported an upward shift of the brain in response to long-duration spaceflight (Roberts et al., 2017). Head-down tilt bed rest (HDBR) also causes a cephalic fluid shift (Hargens and Vico, 2016). Likewise, HDBR has been associated with upward and posterior brain shifts, increased density of brain tissue at the vertex, contraction of adjacent cerebrospinal fluid (CSF) spaces, and increased ventricular volume (Roberts et al., 2015). Collectively, these data suggest that weightlessness provokes fluid and brain shifts, which could be expected to systematically affect EEG recordings.

Here, we present data from two long-duration bed rest studies to identify the time course of resting state electrocortical activity, and the effects of exercise and antioxidant supplementation as countermeasures. The experiments were conducted as part of the European Space Agency (ESA) sponsored 60-days bed rest studies ‘RSL’ and ‘COCKTAIL’. Previous studies investigating the effects of immediate postural changes or short-term head-down tilt (HDT) of up to 2 hours reported decreases in EEG power of the alpha, beta, and gamma frequency bands (Schneider et al., 2008; Chang et al., 2011; Spironelli and Angrilli, 2017). In line with the acute postural effects of HDT, we hypothesized that resting state EEG spectral power would decrease with the onset of the first day of HDBR. Second, we anticipated that functional and structural brain changes observed in response to prolonged HDBR (Zhou et al., 2014; Liao et al., 2015; Yuan et al., 2016, 2018; Friedl-Werner et al., 2020) would result in further changes in EEG spectral power and affect resting state functional connectivity, and that these effects would be moderated by exercise and antioxidant supplementation.

## Methods

### Study Design

#### Experiment 1: Long-Term Effects of HDBR With and Without Exercise as a Countermeasure (RSL)

As part of the ESA sponsored bed rest study ‘Reactive jumps in a Sledge jump system as a countermeasure during Long-term bed rest – RSL Study’ (RSL) we acquired resting state EEG data once before, three times during, and once after 60 days of HDBR to identify the time course of electrocortical activity in response to prolonged HDBR with and without exercise as a countermeasure. The study was carried out at the :envihab facility of the German Aerospace Agency (DLR) in Cologne, Germany in 2015/2016. Details on the general study design and exercise program are described elsewhere (Kramer et al., 2017). Briefly, twenty-three young, healthy right-handed men [age: 29 ± 6 years, height: 181 ± 6 cm, body mass: 77 ± 7 kg (mean ± SD)] with no personal history of neurological or psychiatric illness, drug or alcohol abuse, and normal or corrected-to-normal vision were enrolled in the study. All participants underwent 15 days of baseline data collection (BDC-15 through BDC-1), 60 days of −6 degrees HDBR (HDBR1 through HDBR60) and 15 days of recovery (*R*+0 through *R*+14). On the first day of bed rest, participants were randomly assigned to either an exercise group (RSL-TRAIN, *n* = 12) that performed a high-intensity interval training during HDBR or a control group (RSL-CTRL, *n* = 11) that did not perform any physical training. Each training session consisted of repetitive jumps and different series of countermovement jumps with an average load ≥ 80% of the participant’s body weight. RSL-TRAIN performed a total of 48 exercise sessions during the 60-day bed rest phase (5× per week during the first two weeks of HDBR, and 6× per week for the following six weeks). The sessions were scheduled in the afternoon between 2 pm and 6 pm. Each training had a total duration of 20 min including preparation. Because of medical reasons two participants (one from each group) started their recovery after HDBR49 and HDBR50, respectively (instead of HDBR60). A comparison of the subgroup demographics is displayed in Table 1. There were no significant differences in any subject characteristics (all *p*s > 0.35).

**TABLE 1:** Demographic characteristics for RSL and COCKTAIL subgroups at baseline.*

	RSL		COCKTAIL	
	CTRL	TRAIN	CTRL	TREAT
N	11	12	10	10
Age [years]	28.3 ± 5.5	29.9 ± 6.6	33.5 ± 8.3	34.8 ± 7.5
Height [cm]	179.6 ± 6.5	182.0 ± 5.4	176.1 ± 4.6	176.1 ± 4.7
Body Mass [kg]	77.6 ± 7.5	71.9 ± 5.1	74.9 ± 9.1	73.1 ± 5.7
BMI [kg/m^2^]	23.5 ± 2.1	23.4 ± 1.7	24.1 ± 2.2	23.6 ± 1.6

*Data are means and standard deviations; *N*, sample size; BMI, Body Mass Index. RSL, 60-day head-down tilt bed rest study investigating the effect of bed rest with (TRAIN) and without exercise (CTRL) as a countermeasure (Experiment 1); COCKTAIL, 60-day head-down tilt bed rest study investigating the effect of bed rest with (TREAT) and without (CTRL) antioxidant supplementation as a countermeasure (Experiment 2). There were no significant differences between subgroups within the RSL and Cocktail experiment (all *p*s > 0.35). Participants of the RSL study were slightly but significantly older and taller than participants of the COCKTAIL study (*p* = 0.026 for age and *p* = 0.006 for height).

Resting state eyes-closed EEG data were collected for 3 min seven days prior to bed rest (BDC-7), on the second day of HDBR (HDBR2), on the 28th day of HDBR (HDBR28), on the 56th day of HDBR (HDBR56), and after 11 days of recovery (*R*+10, the first day of recovery was *R*+0). For the two participants that started their recovery earlier, data collection was performed on the last day of their bed rest phase (i.e., HDBR49 and HDBR50, respectively). During the baseline and recovery period data were collected in seated position. During HDBR participants remained in supine position at −6 degrees head-down tilt.

The project was registered with the German Clinical Trials Register (DRKS, registration number DRKS00012946), and approved by the Ethics Committee of the Northern Rhine Medical Association (Ärztekammer Nordrhein) in Düsseldorf, Germany, and the local Ethics Committee of Charité – Universitätsmedizin Berlin, Germany. The study conformed to all standards and ethical principles for medical research on human subjects set out in the Declaration of Helsinki by the World Medical Association. All participants were informed about the purpose, experimental procedures, and risks before giving their verbal and written informed consent to participate in the experiment.

#### Experiment 2: Long-Term Effects of HDBR With and Without Antioxidant/Anti-Inflammatory Supplementation as a Countermeasure (COCKTAIL)

The second experiment was carried out as part of the ESA sponsored bed rest study ‘Effects of a Nutritional Cocktail Consisting of Antioxidant and Anti-inflammatory Supplements to Prevent the Deconditioning Induced by 60 Days of Antiorthostatic Bed Rest’. Resting state EEG data were collected once before, three times during, and once after 60 days of HDBR to identify the time course of electrocortical activity in response to prolonged HDBR with and without an antioxidant/anti-inflammatory nutritional supplement as a countermeasure. The study was carried out at the French Institute for Space Medicine and Physiology (MEDES), Toulouse, France in 2017. Details of the general study design and nutritional supplement are reported elsewhere (Arc-Chagnaud et al., 2020). Briefly, twenty young healthy men (mean age: 34 ± 8 years; mean height: 176 ± 5 cm; mean body mass: 74 ± 7 kg; *n* = 17 right-handed) with no personal history of neurological or psychiatric illness, drug or alcohol abuse, and normal or corrected-to-normal vision were enrolled in the study. The experiment comprised 15 days of baseline data collection (BDC-15 through BDC-1), 60 days of −6 degrees HDBR (HDBR1 through HDBR60) and 15 days of recovery (*R*+0 through *R*+14). On the first day of HDBR, the subjects were randomly allocated to one of two groups. The participants of the treatment group (COCKTAIL-TREAT, *n* = 10) received an antioxidant cocktail, consisting of 741 mg of a bioactive polyphenol compound mix (XXS-2A-BR2 mix, Spiral Company, Dijon, France), 2.1 g omega-3 fatty acids (Omacor, Pierre Fabre Laboratories, Toulouse France), and 138 mg vitamin E coupled with 80 μg of selenium (Solgar, Marne la Vallée, France) during the bed rest phase. The control group (COCKTAIL-CTRL, *n* = 10) did not receive any supplement or other countermeasure. A comparison of demographic group characteristics is displayed in Table 1. There were no significant differences in any subject characteristics (all *p*s > 0.54).

Resting state eyes-closed EEG data were collected for 3 min eight days prior to bed rest (BDC-8), on the seventh day of HDBR (HDBR7), on the 31st day of HDBR (HDBR31), on the 60th day of HDBR (HDBR60), and on the eighth day of the recovery period (*R*+7, the first day of recovery was *R*+0). During baseline and recovery, EEG was recorded in seated position. During HDBR data were collected in supine posture at −6 degrees head-down tilt.

The experiment was registered with the Clinical Trial.gov database under NCT03594799 and approved by the Comité de Protection des Personnes (CPP Sud-Ouest Outre-Mer I), the French Health Authorities (Agence Française de Sécurité Sanitaire des Produits de Santé), and the local Ethics Committee at Charité – Universitätsmedizin Berlin, Germany. The study conformed to all standards and ethical principles for medical research on human subjects set out in the Declaration of Helsinki by the World Medical Association. All participants were informed about the purpose, experimental procedures, and risks before giving their verbal and written informed consent to participate in the experiment.

### Data Acquisition

The measurements from both experiments, i.e., RSL and COCKTAIL, were performed in dimly lit and sound-attenuated rooms in the morning between 8.30 am and 1.30 pm. EEG data were acquired with a 32-channel amplifier (actiCHamp, Brain Products GmbH, Germany). Electrodes were attached to an EEG cap (actiCap, Brain Products GmbH, Germany) at positions Fp1, Fp2, F7, F3, Oz, Fz, F4, F8, FT9, FC5, FC1, TP9, CP5, CP1, TP10, CP6, CP2, FT10, FC6, FC2, FC3, C3, Cz, C4, T7, T8, P7, P3, Pz, P4, P8, O1, and O2, according to the International 10/20 System (Jasper, 1958). Signals were referenced to Fz. Electrode impedance was checked for each subject before data collection and maintained at less than 5 kΩ. Eye movements and eye blinks were monitored via tin electrooculogram (EOG) electrodes (B18 Multitrodes, EASYCAP GmbH, Germany) placed above and below the left eye as well as at the outer canthi of both eyes. EEG and EOG signals were amplified by a multi-channel bio-signal amplifier and A/D converted at 1000 Hz per channel with 24-bit resolution. During the bed rest phase, i.e., when participants were tested in a −6 degrees HDT posture, participants’ heads were placed on a memory foam to minimize discomfort while wearing the EEG cap.

### Data Processing

All data were analyzed offline using EEGLAB (version 2019.1.0), a toolbox embedded in Matlab (version R2015b, The MathWorks, Inc., Natick, Massachusetts, United States). First, the EEG signals were filtered with a 0.5 to 65 Hz bandpass filter. Sinusoidal artifacts (50 Hz line noise) were removed using the CleanLine function (Mullen, 2012). Next, recordings were visually inspected to allow for the interpolation of bad channels. Data from electrodes with poor signal quality were replaced using spherical spline interpolation. On average, less than 2% of the channels had to be interpolated. After re-referencing to average reference, data were segmented into 4096-ms-epochs with an overlap of 10% between consecutive segments. To exclude the possibility that EEG modifications were due to eye movement artifacts or other transient effects related to opening and closing of the eyes, the first and last 5 s of each recording were excluded for the successive analysis. EOG artifacts were removed using vertical and horizontal EOG regression channels (Gómez-Herrero et al., 2007). Muscle artifacts were removed using a spatial filtering framework with defaults (De Clercq et al., 2006). After baseline removal, an automated exclusion procedure was used, rejecting epochs which exceed a gradient threshold of 100 μV, or a maximum and minimum amplitude of ± 200 μV. On average, 89% of the epochs were accepted for further analysis.

Segmented data were analyzed by fast Fourier transform spectral analysis with 0.244 Hz resolution and averaged over all artifact-free epochs to calculate absolute (μV^2^/Hz) power density. For each electrode, the absolute theta (0.5 to 4 Hz), delta (4 to 7.5 Hz), alpha (7.5 to 12.5 Hz), and beta (12.5 to 35.0 Hz) power were exported as the mean of activity values within each frequency band. In agreement with previous work on resting state spectral power (Spironelli and Angrilli, 2017) we clustered the electrodes into four regions of interest with two spatial factors (laterality, region) consisting of two levels each (left/right and anterior/posterior, respectively). Each region comprised the averaged absolute spectral power of six electrodes as follows: (anterior-left) Fp1, F3, FC5, FC1, F7, FT9; (anterior-right) Fp2, F4, FC6, FC2, F8, FT10; (posterior-left) P3, P7, TP9, O1, CP1, CP5; (posterior-right) P4, P8, TP10, O2, CP6, CP2.

Next, we identified the neural sources of resting state electrocortical activity using exact low-resolution brain electromagnetic tomography (eLORETA) (Pascual-Marqui, 2007). eLORETA enables the spatial identification of cortical activity by employing a discrete, three-dimensional distributed, linear, weighted minimum norm inverse solution method that allows for an exact localization to test point sources. We used a three-dimensional head model based on the MNI152 template registered to the Talairach brain atlas and digitized at the Montreal Neurologic Institute (MNI) brain imaging center (Mazziotta et al., 2001). The solution space was limited to the cortical gray matter, including 6239 voxels of 5 mm spatial resolution. All artifact-free EEG epochs were used to calculate the cortical current source density for each of our four frequency bands. The transformed data, containing the corresponding 3D cortical distribution of the electrical neuronal generators were used for further statistical analysis.

We then used eLORETA to analyze the effects of bed rest on source-based functional connectivity of electrocortical activity. In line with previous research on EEG resting state functional connectivity we selected 19 seeds from key regions of the default mode network (DMN) and the fronto-parietal network (Thatcher et al., 2014; Whitton et al., 2018). The MNI coordinates for the seeds are provided in Supplementary Table 1. Given the low spatial resolution of eLORETA (voxel dimension: 5 mm^3^), single voxels that were closest to the seed point were defined as the centroid of each region of interest (ROI). The use of a single ROI voxel reduced the potential bias associated with high correlations among neighboring voxels generated because of the relatively low spatial resolution and inherent smoothness of the eLORETA inverse solution. Connectivity between pairs of all 19 ROIs was then defined as the lagged phase synchronization between the intracortical EEG-source estimates, which is expected to minimize artifacts related to volume conduction and maximize physiological connectivity information (Pascual-Marqui et al., 2011).

### Statistical Analysis

To assess the effects of bed rest and the interventions on EEG spectral power, we performed linear mixed models with participants as random effects (random intercepts), and *Time* (sessions before, during, and after HDBR), *Group* (intervention, control), *Laterality* (left, right), *Region* (anterior, posterior), and their interactions as fixed factors for each frequency band (delta, theta, alpha, beta) and experiment (RSL, COCKTAIL). Covariance matrices were determined by restricted maximum likelihood (REML) estimation. *P*-values were obtained using Satterthwaite’s approximation for denominator degrees of freedom. To assess changes from baseline simple comparisons were performed between the baseline recording before HDBR and all subsequent time points using pre-planned contrasts corrected for multiple comparisons (Hochberg, 1988). Effect sizes were reported as Cohen’s *d* and 95% confidence intervals. The level of significance was set at α = 0.05 (two-sided) for all testing. All statistical analyses and graphical illustrations were carried out using the software package R (version 3.5.1, R Core Team, 2018). Mixed models were run using the packages lme4 and lmerTest (Bates et al., 2015). Estimated marginal means were calculated using emmeans (Lenth, 2016). Figures were created using ggplot2 (Wickham, 2016).

The eLORETA software was used to assess changes in the neural sources of electrocortical activity by performing dependent *t*-tests for log-transformed estimated cortical current density between baseline (before HDBR) and all subsequent time points. Statistical significance was assessed for all frequency bands using a non-parametric randomization procedure with 5000 randomizations that determined the critical probability threshold (*t*_*critical*_) with corrections for multiple testing (Nichols and Holmes, 2002).

To assess changes in EEG resting state functional connectivity between the pairs of the nineteen ROIs in each frequency band, eLORETA was used to perform dependent sample *t*-tests comparing the connectivity values from baseline (before HDBR) with all subsequent points in time. For each of these *t*-tests a total of 684 tests were performed (171 ROI connections x 4 frequency bands). A non-parametric randomization procedure with 5000 randomizations and corrections for multiple testing was used to determine statistical significance (Nichols and Holmes, 2002). The level of significance was set at α = 0.05 (two-sided) for all testing performed using the eLORETA software package.

## Results

### EEG Spectral Power

**FIGURE 1:**
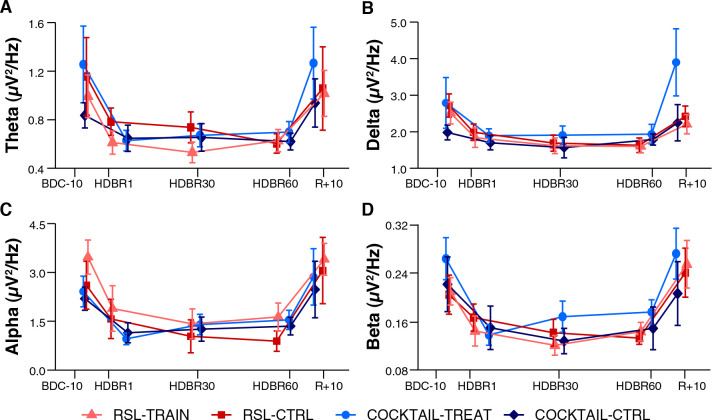
Changes in electrocortical activity during long-duration head-down tilt bed rest of intervention and treatment groups of the RSL and COCKTAIL study. EEG power in **(A)** theta, **(B)** delta, **(C)** alpha, and **(D)** beta frequency bands are averaged over all electrodes (*n* = 31) for RSL-TRAIN (*n* = 11, light red triangle), RSL-CTRL (*n* = 11, dark red square), COCKTAIL-TREAT (*n* = 10, light blue circle), and COCKTAIL-CTRL (*n* = 10, dark blue diamond), respectively. BDC-10 to BDC-1 refer to baseline data collection. HDBR1, HDBR30, and HDBR60 indicate first, 30th, and 60th day of HDBR. R+0 to R+10 correspond to the first and 11th day after HDBR. For RSL data were collected at BDC-7, HDBR2, HDBR28, HDBR56, and R+10. For COCKTAIL data were collected at BDC-8, HDBR7, HDBR30, HDBR60, and R+7. Data are presented as mean and standard errors.

The mean changes in EEG spectral power were very similar between groups (RSL-TRAIN, RSL-CTRL, COCKTAIL-TREAT, and COCKTAIL-CTRL) for all frequency domains (Figure 1). The similarity across studies and intervention and control groups was confirmed by mixed model analyses (see Supplementary Tables 2, 3 for the RSL and COCKTAIL experiment, respectively). There were neither significant main effects for *Group* (all *p*s > 0.441) or *Laterality* (all *p*s > 0.125) nor significant interactions between *Group* and *Laterality, Time*, or *Region* (all *p*s > 0.075) for any frequency band and experiment.

**FIGURE 2:**
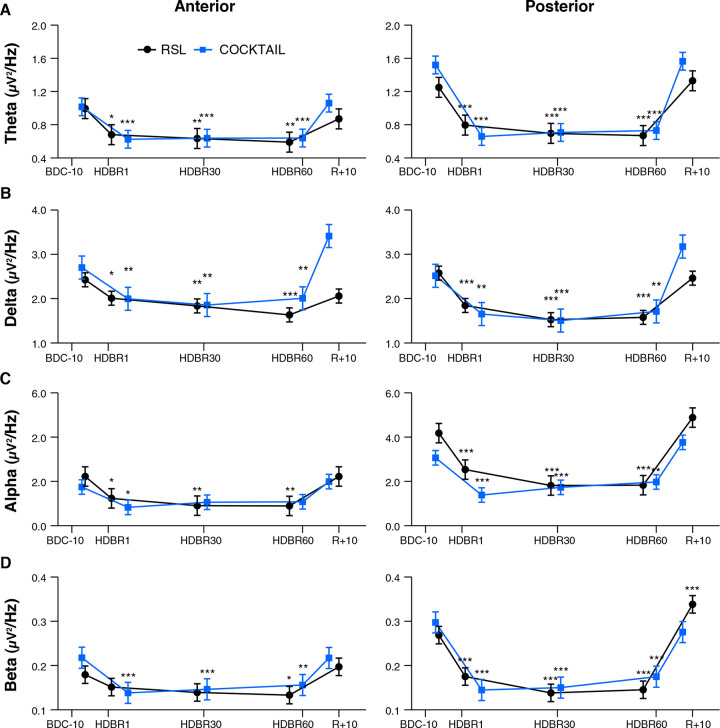
Effect of long-duration head-down tilt bed rest on electrocortical activity. Time courses show changes of EEG spectral power by *Region* (Anterior, Posterior) within the **(A)** theta, **(B)** delta, **(C)** alpha, and **(D)** beta frequency band for RSL (*n* = 23, blue circle), and COCKTAIL (*n* = 20, black square), respectively. Data are presented for each time point as estimated marginal means and standard errors. Significant levels with respect to baseline are indicated by asterisks. BDC-10 to BDC-1 refers to baseline data collection. HDBR1, HDBR30, and HDBR60 indicate first, 30th, and 60th day of HDBR. R+0 to R+10 correspond to the first and 11th day after HDBR. For RSL data were collected at BDC-7, HDBR2, HDBR28, HDBR56, and R+10. For COCKTAIL data were collected at BDC-8, HDBR7, HDBR30, HDBR60, and R+7. *p < 0.05, **p < 0.01, and ***p < 0.001 compared to baseline.

**FIGURE 3:**
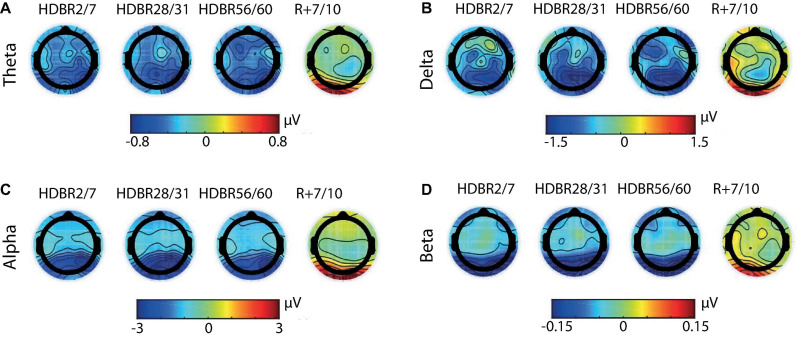
Grand-averaged topographical maps of absolute spectral power within the **(A)** theta, **(B)** delta, **(C)** alpha, and **(D)** beta frequency band. Scalp maps show changes from baseline (BDC-8/-7) to short-term (HDBR2/7), mid-term (HDBR28/31), and long-term (HDBR56/60) head-down tilt bed rest, and recovery (R+7/+10) averaged for all groups (RSL+COCKTAIL, *n* = 43). Absolute power increases are shown in red and decreases in blue. For RSL data were collected at BDC-7, HDBR2, HDBR28, HDBR56, and R+10. For COCKTAIL data were collected at BDC-8, HDBR7, HDBR30, HDBR60, and R+7. BDC, baseline data collection; HDBR, head-down tilt bed rest; R, recovery.

Irrespective of the experiment and subgroup mean EEG spectral power decreased after the onset of HDBR, remained decreased during HDBR, and returned to baseline levels after the cessation of HDBR. This pattern was quantified by significant main effects for *Time* and *Region*, and a significant interaction between *Time* and *Region*. These effects were observed for all frequency bands except for delta power in the COCKTAIL study (*Time* x *Region*: *F*_4,342_ = 0.10, *p* = 0.983). Figure 2 shows the time courses of absolute EEG spectral power by *Region* (Anterior, Posterior) within the theta, delta, alpha, and beta frequency band for both experiments (RSL, COCKTAIL). Details on the effects of *Time* by *Region* (Anterior, Posterior) for each frequency band (theta, delta, alpha, beta) and experiment (RSL, COCKTAIL) are provided in Supplementary Tables 4, 5. Briefly, contrasts (*Time* by *Region*) revealed that all power indices significantly decreased during the bed rest period at posterior sites, reaching a plateau as early as at the first measurement during HDBR, i.e., 24 h of bed rest for RSL and 7 days of bed rest for COCKTAIL. A similar though less pronounced pattern was observed for the anterior region. Spectral power significantly decreased during HDBR within the delta, theta, and alpha frequency ranges for RSL, and within the delta, theta, and beta frequency domain for COCKTAIL. In both regions the reductions in EEG spectral power returned to baseline after the cessation of bed rest. Figure 3 displays the topographical distributions pooled for both experiments (RSL and COCKTAIL). The topographical maps indicate that the decrease in absolute power during HDBR was related to a decrease in spectral power across all electrode sites with larger reductions at posterior areas of the brain. Visual inspection did not reveal any effect of *Time* between short-, mid- and long-term HDBR. This was confirmed by a mixed model ANOVA yielding no significant interaction of *Time* and *Region* for any of the investigated frequency bands (all *p*s > 0.582 for RSL; all *p*s > 0.615 for COCKTAIL) when including HDBR data only. To account for inter-individual and intra-individual differences we z-transformed the absolute power values across participants and testing days and re-analyzed the data set. The analyses confirmed the previous findings. EEG spectral power significantly decreased after the beginning of HDBR, remained decreased during HDBR, and reached baseline levels after the completion of bed rest (see Supplementary Figure 1).

### eLORETA Source Localization

Table 2 summarizes the results for the analyses of the neural bases of electrocortical activity in response to HDBR. In line with the analyses performed on spectral power, we assessed changes in cortical current density between testing days irrespective of the subgroups (intervention and control) of each experiment.

**TABLE 2:** Contrasts indicating differences in eLORETA cortical current density between baseline (BDC-8 for COCKTAIL and BDC-7 for RSL) and all subsequent points in time.*

Experiment	Study Day	*t*_*critical*_	Alpha		Beta	
			*t*_*max*_	x y z	*t*_*max*_	x y z
RSL	HDBR2	4.48	–4.96	30 −85 15	–5.83	15 −65 10
	HDBR28	4.57	–5.77	20 −65 30	–7.96	−5 −65 5
	HDBR56	4.57	–4.70	45 −45 35	–6.989	5 −60 15
	R+10	4.55	3.84	−15 −10. −15	3.24	−45 −65 45
COCKTAIL	HDBR7	4.50	–6.18	45 −35 35	–7.54	20 −70 20
	HDBR31	4.65	–6.41	45 −5 35	–8.11	10 −65 20
	HDBR60	4.58	–5.04	55 −5 35	–9.60	20 −80 20
	R+7	4.67	4.22	−40 −5 0	–2.49	35 15 0

*RSL, 60-day head-down tilt bed rest study investigating the effect of bed rest with and without exercise as a countermeasure (*n* = 23, Experiment 1); COCKTAIL, 60-day head-down tilt bed rest study investigating the effect of bed rest with and without antioxidant supplementation as a countermeasure (*n* = 20, Experiment 2); HDBR, head down-tilt bed rest; R, recovery; *t*_*critical*_, critical probability threshold of non-parametric randomization test with 5000 randomizations corrected for multiple comparisons; *t*_*max*_, maximal *t-*statistic*; x, y, z*, MNI coordinates of peak voxel.

**FIGURE 4:**
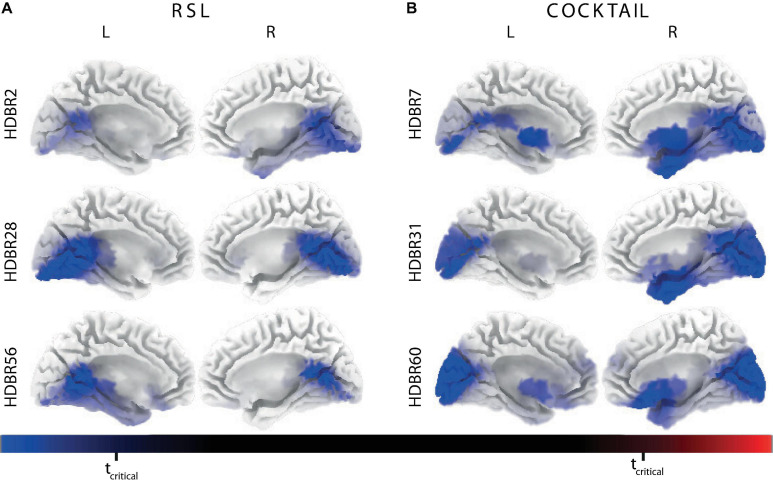
Statistical parametric maps (SPMs) indicating significant effects of *Time* using the baseline (before bed rest) current source density distribution as a reference on brain source localization for **(A)** RSL (*n* = 23) and **(B)** COCKTAIL (*n* = 20) within the beta frequency domain. The color scale displays *t*-values with blue colors indicating decreased activity during head-down tilt bed rest (HDBR) compared to baseline and red colors indicating increased activity. All cortical regions showing significant effects are listed in Supplementary Table 6. L, left hemisphere; R, right hemisphere; *t*_*critical*_, critical probability threshold of non-parametric randomization tests with 5000 randomizations corrected for multiple comparisons.

We found significantly lower cortical activations within the alpha and beta frequency band on HDBR2, HDBR28, and HDBR56 compared to BDC-7 in the RSL experiment (all *t*s > 4.48, all *p*s < 0.05). Likewise, we observed statistically lower cortical activations within the alpha and beta frequency ranges between BDC-8 and HDBR7, HDBR31, and HDBR60 in the COCKTAIL study (all *t*s > 4.50, all *p*s < 0.05). As shown in Figure 4 the inhibition of electrocortical activity during HDBR was localized in a broad cluster of voxels, including but not limited to the bilateral precuneus, posterior cingulate gyrus, and lingual gyrus. This effect was very similar in both studies, frequency domains, and across time points. A list of cortical regions showing significant effects is provided in Supplementary Table 6.

Visual inspection also revealed reductions in cortical current density during HDBR within the delta and theta domain, but these effects did not reach statistical significance. We also did not find any significant differences between data collected during the baseline and recovery periods (*t*_*max*_ = 3.84, *p* = 0.163 for RSL; *t*_*max*_ = 4.22, *p* = 0.099 for COCKTAIL) and between data collected during the different HDBR testing sessions (all *t*s < 3.98, all *p*s > 0.167).

### eLORETA Functional Connectivity

The results of the functional connectivity analyses are summarized in Supplementary Table 7. Briefly, we did not observe any changes in functional connectivity between baseline (before bed rest) and all subsequent time points for any of the frequency bands and experiments (all *t*s < 3.86 and all *p*s > 0.141). We also did not find a significant difference between data collected during the HDBR sessions (all *t*s < 3.95 and all *p*s > 0.148).

## Discussion

This study aimed to identify the time course of resting state electrocortical activity in response to prolonged HDBR using data from two 60-day bed rest studies conducted at two different sites. Our data revealed a considerable and significant decrease in EEG spectral power across all frequency bands during HDBR. Likewise, we demonstrated significantly lower activity of the neural sources of electrocortical activity within the alpha and beta frequency domain over a wide range of brain regions. These changes occurred immediately after the onset of HDBR, i.e., after 24 h of bed rest, and were completely uncoupled from the duration of bed rest. Prolonged bed rest did not induce any further changes in resting EEG spectral power or cortical source distribution of resting state EEG. After the cessation of HDBR electrocortical activity was not significantly different from baseline levels recorded before HDBR. The time courses of EEG spectral power and cortical source distribution were highly comparable between the RSL and COCKTAIL study, and neither exercise nor antioxidant supplementation as a countermeasure affected this response.

Our results of the immediate effects of HDBR on EEG spectral power are in line with various data previously published on the acute effects of supine or HDT position on absolute power and cortical source distribution of resting state EEG, reporting decreases within high-frequency domains including alpha and beta power (Schneider et al., 2008; Chang et al., 2011; Thibault et al., 2014; Spironelli and Angrilli, 2017). Based on MRI studies accounting functional and structural brain changes in response to prolonged bed rest (Rao et al., 2014; Zhou et al., 2014; Roberts et al., 2015; Yuan et al., 2016; Friedl-Werner et al., 2020), we also expected alterations in resting state EEG after 30 days and 60 days of bedrest. In contrast to this hypothesis, we could not demonstrate any changes in EEG spectral power or EEG resting state functional connectivity with increasing duration of HDBR. These findings suggest that the reductions in electrocortical activity observed in our study can be attributed to postural changes rather than the immobilization associated with long-term bed rest.

Several mechanisms are likely to have contributed to the effects of HDT on electrical scalp activity. HDT has been shown to modulate brain hemodynamics by increasing cerebral blood flow. Kawai et al. (2003) and Kurihara et al. (2003) reported elevations in brain oxygenation and hemoglobin concentrations in HDT position. The relationship between local neural activity and changes in cerebral blood flow has been well established (Shibasaki, 2008; Chiarelli et al., 2017). A number of studies have shown that alterations in cerebral oxygenation are associated with changes in electrocortical power (Pfurtscheller et al., 2012; Lachert et al., 2017; Dravida et al., 2019; Lin et al., 2020). For instance, Pfurtscheller et al. (2012) reported a coupling between prefrontal oxyhemoglobin (HbO_2_) and central EEG alpha and beta power. Further evidence comes from Lachert et al. (2017) showing that increases in cortical HbO_2_ concentration are related to decreases in alpha and beta power during a motor task. It is therefore possible that changes in brain hemodynamics during HDT demonstrated by Kawai et al. (2003) and Kurihara et al. (2003) are accompanied by a modulation of electrocortical activity. Similar conclusions were also reached by Schneider et al. (2008) who attributed decreases in alpha and beta power during supine and −6 degrees HDT position to an increase in brain oxygenation and hemoglobin saturation (Schneider et al., 2008). These assumptions were questioned by Lipnicki (2009) who proposed an alternative explanation associated with the interaction between the brain and the autonomous control of the cardiovascular system (Lipnicki, 2009). Postural changes induce a cephalic fluid shift that leads to increases in thoracic blood volume and hydrostatic pressure, stimulating cardiopulmonary and arterial baroreceptors, which in turn reduce sympathetic system activation (Mohrman and Heller, 2018). There is ample evidence that arterial baroreceptor stimulation inhibits cortical activity (Rau et al., 1993). This effect seems to be mediated by decreasing locus coeruleus activity and cortical noradrenaline turnover (Murase et al., 1994; Berridge and Waterhouse, 2003), which are key modulators of arousal and wakefulness. As noted by Elam et al. (1984) postural changes from upright to supine may dampen arousal via reduced locus coeruleus-noradrenergic system activity in response to increased baroreceptor stimulation.

Another explanation for the global decrease in electrocortical activity seen in the present study may be attributed to the HDT-induced shift of the brain together with a change in cerebrospinal fluid (CSF) layer thickness and a redistribution of CSF. Alperin et al. (2005) investigated the acute effects of postural changes from sitting upright to supine on CSF using CSF flow imaging (Alperin et al., 2005). They observed that intracranial CSF volume increases from sitting to supine position. Simulation studies have shown that minute shifts in CSF concentration can have considerable effects on EEG signals (Ramon et al., 2004; Wendel et al., 2008; Akalin Acar and Makeig, 2013). CSF is up to ten times more conductive than white or gray matter, and up to 100 times more conductive than bone (Ramon et al., 2006). Despite CSF being highly conductive, it weakens the electric field and current density in the scalp (Stenroos and Nummenmaa, 2016). The role of CSF on electrocortical activity has also been illustrated experimentally by measuring EEG in prone and supine position (Rice et al., 2013). Rice et al. (2013) reported an inverse relationship between CSF layer thickness and electrocortical power. They attributed these changes to instant shifts in CSF thickness associated with reallocations of the brain within the skull as a result of the different head orientations. Results from prolonged bed rest studies do not show indications for an overall fluid increase within the intracranial compartment, but rather a redistribution of existing CSF (Roberts et al., 2015; Koppelmans et al., 2017). This was shown by Roberts et al. (2015) who observed an upward shifting of the center’s brain mass concomitant with a posterior rotation of the brain relative to the skull of less than 1 mm during HDBR. Such a brain shift is considerably smaller than the electrode location precision typically obtained for EEG recordings. The uncertainty of electrode positions relative to the cortex can be expected to be within several millimeters for highly trained operators. Notably, even small variations in electrode positions can lead to significant shifts in estimated source localizations. For instance, a change in electrode position of 1 cm could lead to a shift of a single dipole by more than 2 cm (Shirazi and Huang, 2019). However, even considering such uncertainties, they would not contradict the possibility that the upward brain shift and the reallocation of CSF associated with HDBR could have caused the attenuation of the EEG signal observed in the present study.

Our findings are also consistent with observations made during acute exposure to microgravity, showing a global decrease of electrocortical activity with the onset of weightlessness (Schneider et al., 2008; Klein et al., 2019). In contrast, early studies on EEG recordings during spaceflight reported no changes (Maulsby, 1966) or increases in alpha, theta and beta power (Frost et al., 1975, 1977). Cheron et al. (2006) used one-minute resting state recordings alternating between eyes opened and eyes closed every ten seconds to assess the impact of long-duration spaceflight on event-related spectral perturbations. They reported increases in alpha power during the arrest reaction in eyes-closed state (Cheron et al., 2006) compared to pre-mission levels. Recently, Cebolla et al. (2016) employed a normalized measure on resting EEG data collected before administering a visuo-attention task to investigate the effect of microgravity, and observed decreases in alpha power desynchronization (Cebolla et al., 2016) in relation to pre-mission. According to the authors the changes observed during/after long-term space missions can be explained by an increased demand for the integration and processing of vestibular information due to the decreased gravitational reference frame in space as well as by the reduction of support related proprioceptive afferents. As bed rest is not a simulation of microgravity but mimics some of the physiological responses to weightlessness these data may not directly translate the data of the present experiments. Additionally, the use of different methodologies could account for the observed discrepancies. Similar to a recent proposition for standardizing brain imaging protocols for spaceflight (Roberts et al., 2020), normative data on EEG recordings using a set of standardized procedures and analyses could help elucidating the effects related to fluid and brain shifts vs. possible structural and functional reorganization of the brain during prolonged spaceflight and spaceflight analogs.

Although the study was highly controlled, our findings are subject to a few limitations. First, EEG signals are prone to physiological (e.g., ocular and muscle activity) and non-physiological artifacts (e.g., electromagnetic interferences and electrode artifacts) that may affect the reliability of the data (Tandle et al., 2015). By using hardware equipped with active noise cancellation and electrodes that amplify the signal directly at the recording site (active circuits for impedance conversion were integrated directly in the electrodes); standardizing the data collection procedures; instructing participants not to move; recording EOG data; and employing rigorous and robust pre-processing pipelines including visual inspection, filtering, and artifact rejection, we minimized the impact of noise on EEG recordings. However, even under consideration of excellent signal-to-noise ratios, cephalic fluid shifts associated with postural changes (or changing gravity levels) raise caution regarding the interpretability of EEG recordings in these conditions. Specifically, the electric field and current flow in the scalp are considerably attenuated by the CSF and the resistive properties of the skull (Van Den Broek et al., 1998). We also acknowledge that EEG lacks the spatial resolution for identifying sub-cortical structures that could be critical for operational performance during spaceflight. A mathematical approach for representing current source density of EEG recordings in 3D space is eLORETA, which we also employed in the current study. Several studies have confirmed that eLORETA has zero localization error in the presence of measurement and structured biological noise (Dattola et al., 2020). It should be noted though that eLORETA relies on a standard head model that does not account for interindividual variability in brain size and shape as well as tissue conductivity that can affect localization accuracy. In addition, EEG measures of brain connectivity can be confounded by volume conduction effects (He et al., 2019). We tried to minimize these effects by employing source localization-based connectivity measure. However, due to the high correlation of adjacent voxels and the relatively low spatial resolution and inherent smoothness of the eLORETA inverse solution our findings should be interpreted with caution. Finally, data were collected in −6 degrees HDT during the bed rest phase. In contrast, participants were tested in seated position during the baseline and recovery period, resulting in global reductions of electrocortical power. It is therefore possible that the filtering effects associated with postural changes have masked underlying bed rest related alterations. Future studies should therefore employ the same position on all measurement days to discriminate the impact of bed rest from the effects of posture.

Taken together, our data show that HDBR reduces electrocortical activity and its neural sources within a broad range of brain regions. These changes occur as early as after 24 h of HDBR and can be expected to onset immediately after bed rest commencement. The reductions in EEG spectral power and cortical source distribution persist until returning to an upright position again after the cessation of bed rest. Considering previous studies using structural brain imaging we attribute the alterations in EEG power to a brain shift and redistribution of CSF in response to the postural change to HDT. Our findings offer a plausible mechanism for EEG changes observed during bed rest, and should be taken into consideration in the presence of cephalic fluid shifts. Furthermore, prolonged bed rest, i.e., increasing time in HDBR did not result in further changes of EEG spectral power and cortical source distribution, suggesting that immobilization and inactivity *per se* do not affect resting state electrocortical activity during HDBR. These findings raise some caution about the use of resting state EEG recordings to identify the time course of changes in brain function during prolonged HBDR. Future bed rest studies employing EEG should consider the use of −6 degrees supine position for all recordings, i.e., including the baseline and recovery period, allow sufficient time to adapt to the postural change minimizing the effects associated with fluid shifts, and also acquire event-related task data or event-related spectral perturbations to identify the effects of prolonged bed rest on electrocortical changes and performance. Likewise, our findings could also have important implications for EEG resting state data collections performed during spaceflight or altered gravity conditions. For instance, to determine the time course of resting state EEG during spaceflight, early inflight recordings could serve as a baseline for follow-up data collections. Future studies may also be able to systematically validate the effects of brain shifts and redistribution of CSF at varying levels of gravity on resting EEG recordings, which could provide the basis to apply normalization techniques to EEG recordings performed under microgravity conditions. Collectively, such approaches could help to disentangle the neurobehavioral impact of spaceflight stressors from cephalic fluid and brain shifts, and reveal the full potential of resting state EEG recordings during human space exploration.

## Data Availability Statement

The datasets presented in this study can be found in online repositories. The names of the repository/repositories and accession number(s) can be found below: http://doi.org/10.6084/m9.figshare.12148359.

## Ethics Statement

The studies involving human participants were reviewed and approved by Ethics Committee of the Northern Rhine Medical Association (Ärztekammer Nordrhein) in Düsseldorf, Germany, the Comité de Protection des Personnes (CPP Sud-Ouest Outre-Mer I), the French Health Authorities (Agence Française de Sécurité Sanitaire des Produits de Santé), and the Ethics Committee of Charité – Universitätsmedizin Berlin, Germany. The participants provided their written informed consent to participate in this study.

## Author Contributions

AS conceived, designed, planned, and supervised the experiments. KB collected the data for Experiment 1, and AF-W for Experiment 2. KB processed the data. KB and AS performed statistical analysis and wrote the manuscript. AF-W, H-CG, and MM provided critical feedback and contributed to the interpretation of the results. All authors discussed the results and reviewed the manuscript.

## Conflict of Interest

The authors declare that the research was conducted in the absence of any commercial or financial relationships that could be construed as a potential conflict of interest.
