# Supplementary material for: Head-Down Tilt Position, but Not the Duration of Bed Rest Affects Resting State Electrocortical Activity
Source: Front Physiol. 2021 Feb 24;12:638669. doi: 10.3389/fphys.2021.638669 (PMC7951060; doi:10.3389/fphys.2021.638669)
Supplement: Supplementary file 1 [file Data_Sheet_1.pdf]

SUPPLEMENTARY MATERIAL

Supplementary Table 1: Seed coordinates used for functional connectivity analysis.*

**Anatomical Structure**	**MNI coordinates**		
	x	y	z
Left posterior inferior parietal lobule	-50	-70	30
Right posterior inferior parietal lobule	50	-70	30
Left anterior inferior parietal lobule	-50	-50	45
Right anterior inferior parietal lobule	50	-50	45
Left hippocampal formation	-20	-20	-20
Right hippocampal formation	20	-20	-20
Left superior parietal lobule	-25	-50	60
Right superior parietal lobule	25	-50	60
Anterior cingulate cortex	5	30	25
Left anterior insula	-30	20	5
Right anterior insula	30	20	5
Posterior cingulate cortex	0	-55	15
Left medial frontal cortex	-35	55	5
Right medial frontal cortex	35	55	5
Left parahippocampal gyrus	-25	-25	20
Right parahippocampal gyrus	25	-25	20
Precuneus	0	-75	45
Left middle temporal gyrus	-65	-20	-10
Right middle temporal gyrus	65	-20	-10

*The eLORETA solution space was restricted to the cortical gray matter of a realistic head model (MNI152), co-registered to the Talairach brain atlas and digitized at the Montreal Neurologic Institute (MNI) brain imaging center. A single voxel that was closest to the seed point was defined as the centroid of each region of interest (ROI).

Supplementary Table 2: Mixed-models of spectral analysis assessing the effects of *Time*, *Group, Region, Laterality,* and their interaction for the RSL study (n = 23).*

**Effect**	**Theta**				**Delta**				**Alpha**				**Beta**			
	df_1	df_2	F	p	df_1	df_2	F	p	df_1	df_2	F	p	df_1	df_2	F	p
Time	4	399	20.54	<0.001	4	399	23.50	< 0.001	4	399	31.38	< 0.001	4	399	40.58	< 0.001
Group	1	21	0.41	0.530	1	21	0.40	0.533	1	21	0.59	0.891	1	21	0.02	0.891
Region	1	399	16.69	<0.001	1	399	0.01	0.967	1	399	87.62	< 0.001	1	399	43.80	< 0.001
Laterality	1	399	0.01	0.936	1	399	0.01	0.974	1	399	0.68	0.206	1	399	1.61	0.206
Time x Group	4	399	1.04	0.383	4	399	0.18	0.949	4	399	0.46	0.375	4	399	1.06	0.375
Time x Region	4	399	2.45	0.046	4	399	3.15	0.014	4	399	0.46	< 0.001	4	399	11.29	< 0.001
Group x Region	4	399	0.05	0.821	4	399	4.45	0.035	4	399	1.67	0.447	4	399	0.58	0.447
Time x Laterality	1	399	0.24	0.916	1	399	0.31	0.870	1	399	0.09	0.931	1	399	0.25	0.931
Group x Laterality	1	399	0.26	0.611	1	399	0.89	0.345	1	399	0.79	0.643	1	399	0.21	0.643
Region x Laterality	1	399	0.71	0.399	1	399	0.21	0.647	1	399	3.49	0.484	1	399	0.49	0.484
Time x Group x Region	4	399	0.07	0.992	4	399	0.38	0.821	4	399	0.22	0.819	4	399	0.39	0.819
Time x Group x Laterality	4	399	0.01	0.999	4	399	0.26	0.905	4	399	0.02	0.963	4	399	0.15	0.963
Time x Region x Laterality	4	399	0.51	0.729	4	399	0.44	0.781	4	399	0.10	0.881	4	399	0.30	0.881
Group x Region x Laterality	1	399	0.10	0.755	1	399	0.01	0.942	1	399	0.91	0.737	1	399	0.11	0.737
Time x Region x Group x Laterality	4	399	0.17	0.952	4	399	0.25	0.910	4	399	0.09	0.988	4	342	0.08	0.988

*Mixed-models were performed separate for each frequency band using *Time*, *Group*, *Region*, and *Laterality* as fixed factors and subject as a random factor. df_1, numerator degrees of freedom; df_2, denominator degrees of freedom; F, F-statistics, p, p-value.

Supplementary Table 3: Mixed-models of spectral analysis assessing the effects of *Time*, *Group, Region, Laterality,* and their interaction for the COCKTAIL study (n = 20).*

Effect	Theta				Delta				Alpha				Beta			
	df_1	df_2	F	p	df_1	df_2	F	p	df_1	df_2	F	p	df_1	df_2	F	p
Time	4	342	60.89	< 0.001	4	342	36.61	< 0.001	4	342	21.45	< 0.001	4	18	41.21	< 0.001
Group	1	18	0.35	0.562	1	18	0.23	0.636	1	18	0.09	0.762	1	342	0.62	0.441
Region	1	342	37.53	< 0.001	1	342	7.88	0.005	1	342	52.44	< 0.001	1	342	18.60	< 0.001
Laterality	1	342	0.28	0.597	1	342	0.61	0.436	1	342	0.24	0.623	1	342	0.13	0.719
Time x Group	4	342	1.49	0.204	4	342	1.20	0.310	4	342	0.49	0.742	4	342	2.37	0.092
Time x Region	4	342	7.58	< 0.001	4	342	0.10	0.983	4	342	2.42	0.048	4	342	3.80	0.005
Group x Region	4	342	0.46	0.497	4	342	3.20	0.075	4	342	0.06	0.808	4	342	0.01	0.928
Time x Laterality	1	342	0.16	0.957	1	342	0.31	0.862	1	342	0.14	0.970	1	342	0.76	0.551
Group x Laterality	1	342	0.35	0.553	1	342	0.94	0.333	1	342	0.35	0.554	1	342	2.36	0.125
Region x Laterality	1	342	0.23	0.629	1	342	0.01	0.938	1	342	2.96	0.086	1	342	0.86	0.353
Time x Group x Region	4	342	0.14	0.967	4	342	0.39	0.813	4	342	0.06	0.994	4	342	0.29	0.886
Time x Group x Laterality	4	342	0.62	0.648	4	342	0.68	0.606	4	342	0.08	0.988	4	342	0.81	0.518
Time x Region x Laterality	4	342	0.10	0.982	4	342	0.22	0.927	4	342	0.29	0.881	4	342	0.40	0.812
Group x Region x Laterality	1	342	0.25	0.617	1	342	1.95	0.163	1	342	0.71	0.398	1	342	0.25	0.615
Time x Region x Group x Laterality	4	342	0.25	0.909	4	342	0.16	0.958	4	342	0.15	0.962	4	342	0.40	0.805

*Mixed-models were performed separate for each frequency band using *Time*, *Group*, *Region*, and *Laterality* as fixed factors and subject as a random factor. df_1, numerator degrees of freedom; df_2, denominator degrees of freedom; F, F-statistics, p, p-value.

Supplementary Table 4. Contrasts examining the effect of *Time* on spectral power for the RSL study (n = 23) using baseline as a reference level.*

Frequency Band	Time	Region	*df*	*t*	p_{corr}	*Effect Size d (95% CI)*
**Theta**	HDBR2	Anterior	399	-2.96	0.013	-1.23 (-2.12, -0.32)
	HDBR2	Posterior	399	-4.27	< 0.001	-1.78 (-2.75, -0.79)
	HDBR28	Anterior	399	-3.38	0.003	-1.41 (-2.32, -0.48)
	HDBR28	Posterior	399	-5.21	< 0.001	-1.78 (-3.2, -1.11)
	HDBR56	Anterior	399	-3.8	0.001	-1.59 (-2.52, -0.62)
	HDBR56	Posterior	399	-5.46	< 0.001	-2.28 (-3.33, -1.2)
	R+10	Anterior	399	-1.16	0.981	0.49 (-1.31, 0.35)
	R+10	Posterior	399	0.75	1	0.31 (-0.52, 1.13)
**Delta**	HDBR2	Anterior	399	-2.64	0.034	-1.1 (-1.97, -0.21)
	HDBR2	Posterior	399	-4.64	< 0.001	-1.94 (-2.92, -0.92)
	HDBR28	Anterior	399	-3.75	0.001	-1.56 (-2.49, -0.61)
	HDBR28	Posterior	399	-6.68	< 0.001	-2.79 (-3.94, -1.6)
	HDBR56	Anterior	399	-5.03	< 0.001	-2.1 (-3.12, -1.05)
	HDBR56	Posterior	399	-6.35	< 0.001	-2.65 (-3.77, -1.49)
	R+10	Anterior	399	-2.33	0.081	-0.97 (-1.83, -0.09)
	R+10	Posterior	399	-0.72	1	-0.3 (-1.12, 0.52)
**Alpha**	HDBR2	Anterior	399	-2.67	0.032	-1.11 (-1.98, -0.22)
	HDBR2	Posterior	399	-4.44	< 0.001	-1.85 (-2.82, -0.85)
	HDBR28	Anterior	399	-3.55	0.002	-1.48 (-2.4, -0.54)
	HDBR28	Posterior	399	-6.38	< 0.001	-2.66 (-3.79, -1.5)
	HDBR56	Anterior	399	-3.59	0.002	-1.5 (-2.41, -0.55)
	HDBR56	Posterior	399	-6.34	< 0.001	-2.64 (-3.77, -1.49)
	R+10	Anterior	399	0.00	1	0 (-0.82, 0.82)
	R+10	Posterior	399	1.89	0.236	0.79 (-0.07, 1.63)
**Beta**	HDBR2	Anterior	399	-1.56	0.482	-0.65 (-1.48, 0.2)
	HDBR2	Posterior	399	-5.22	< 0.001	-2.18 (-3.21, -1.11)
	HDBR28	Anterior	399	-2.24	0.103	-0.93 (-1.79, -0.06)
	HDBR28	Posterior	399	-7.28	< 0.001	-2.04 (-4.24, -1.79)
	HDBR56	Anterior	399	-2.57	0.042	-1.07 (-1.94, -0.18)
	HDBR56	Posterior	399	-6.88	< 0.001	-2.87 (-4.04, -1.66)
	R+10	Anterior	399	0.99	1	0.41 (-0.42, 1,24)
	R+10	Posterior	399	3.88	< 0.001	1.62 (0.65, 2.55)

*Data show effects of *Time* (HDBR2, HDBR28, HDBR56, R+10) by *Region* (Anterior, Posterior) using baseline (BDC-7) as a reference. *df*, degrees of freedom; $p_{corr.}$, p-value corrected for multiple comparisons using the Bonferroni correction for each main effect (theta, delta, alpha, and beta power); Effect Size is Cohen's d; 95% CI, 95% confidence interval.

Supplementary Table 5. Contrasts examining the effect of *Time* on spectral power for the COCKTAIL study (n = 20) using baseline as a reference level.*

Frequency Band	Time	Region	df	t	p_{corr}	Effect Size d (95% CI)
Theta	HDBR7	Anterior	342	-4.45	< 0.001	-1.86 (-2.83, -0.85)
	HDBR7	Posterior	342	-9.82	< 0.001	-4.09 (-5.55, -2.6)
	HDBR31	Anterior	342	-4.31	< 0.001	-1.80 (-2.76, -0.8)
	HDBR31	Posterior	342	-9.26	< 0.001	-3.86 (-5.27, -2.43)
	HDBR60	Anterior	342	-4.28	< 0.001	-1.79 (-2.75, -0.79)
	HDBR60	Posterior	342	-9.00	< 0.001	-3.75 (-5.13, -2.35)
	R+7	Anterior	342	0.51	1	0.21 (-0.61, 1.03)
	R+7	Posterior	342	0.50	1	0.21 (-0.61, 1.03)
Delta	HDBR7	Anterior	342	-3.11	0.008	-1.30 (-2.19, -0.38)
	HDBR7	Posterior	342	-3.81	0.001	-1.59 (-2.52, -0.63)
	HDBR31	Anterior	342	-3.73	0.001	-1.56 (-2.49, -0.6)
	HDBR31	Posterior	342	-4.46	< 0.001	-1.86 (-2.84, -0.86)
	HDBR60	Anterior	342	-3.81	0.009	-1.28 (-2.17, -0.36)
	HDBR60	Posterior	342	-3.56	0.002	-1.48 (-2.40, -0.54)
	R+7	Anterior	342	3.14	0.007	1.31 (0.39, 2.2)
	R+7	Posterior	342	2.91	0.015	1.21 (0.31, 2.10)
Alpha	HDBR7	Anterior	342	-2.85	0.019	-1.19 (-2.07, -0.28)
	HDBR7	Posterior	342	-5.22	< 0.001	-2.18 (-3.21, -1.11)
	HDBR31	Anterior	342	-2.13	0.135	-0.89 (-1.74, -0.02)
	HDBR31	Posterior	342	-4.14	< 0.001	-1.73 (-2.68, -0.74)
	HDBR60	Anterior	342	-2.07	0.157	-0.86 (-1.71, -0.01)
	HDBR60	Posterior	342	-3.39	0.003	-1.41 (-2.32, -0.48)
	R+7	Anterior	342	0.76	1	0.32 (-0.51, 1.13)
	R+7	Posterior	342	2.17	0.124	0.9 (0.03, 1.76)
Beta	HDBR7	Anterior	342	-4.58	< 0.001	-1.91 (-2.89, -0.89)
	HDBR7	Posterior	342	-8.79	< 0.001	-3.67 (-5.02, -2.28)
	HDBR31	Anterior	342	-4.11	< 0.001	-1.71 (-2.67, -0.73)
	HDBR31	Posterior	342	-8.48	< 0.001	-3.54 (-4.86, -2.18)
	HDBR60	Anterior	342	-3.55	0.002	-1.48 (-2.4, -0.54)
	HDBR60	Posterior	342	-7.06	< 0.001	-2.95 (-4.13, -1.72)
	R+7	Anterior	342	-0.04	1	-0.01 (-0.83, 0.8)
	R+7	Posterior	342	-1.26	0.835	-0.53 (-1.35, 0.31)

*Data show effects of *Time* (HDBR7, HDBR31, HDBR60, R+7) by *Region* (Anterior, Posterior) using baseline (BDC-8) as a reference. *df*, degrees of freedom; $p_{corr.}$, *p*-value corrected for multiple comparisons using the Bonferroni correction for each main effect (theta, delta, alpha, and beta power); Effect Size is Cohen's *d*; 95% CI, 95% confidence interval.

Supplementary Table 6. Number of significant voxels ($p < 0.05$) per lobe and hemisphere revealed by eLORETA analysis for the RSL (N = 23) and COCKTAIL study ($n = 20$) showing decreases in alpha and beta cortical current density with respect to baseline.[*]

	**Brain Region**	**Short-term**				**Mid-term**				**Long-term**			
		Alpha		Beta		Alpha		Beta		Alpha		Beta	
		L	R	L	R	L	R	L	R	L	R	L	R
**RSL**	Frontal lobe				4	1	1					7	
	Limbic Lobe	10	20	32	74	74	61	109	59	3	2	110	36
	Occipital Lobe	10	100	22	207	217	163	268	236	1	9	78	49
	Parietal lobe	39	53	7	11	117	123	20	23	12	26	2	3
	Sub-lobar				3	1	1	13	5	2	14	5	2
	Temporal lobe		8		108	8	27	109	11	10	1	27	
**COCKTAIL**	Frontal lobe		1	3	192				166			23	172
	Limbic Lobe	24	23	49	187	2	6	18	136	8	18	31	78
	Occipital Lobe			59	336	11	27	114	351	6	11	207	259
	Parietal lobe		146	9	90	27	245	10	65	23	155	27	57
	Sub-lobar		12		100		19		48		15		26
	Temporal lobe		4		557		50		505		60	1	152

[*]The eLORETA solution space was restricted to the cortical gray matter of a realistic head model (MNI152) registered to the Talairach brain atlas. Short-term, mid-term, and long-term refer to HDBR2/HDBR7, HDBR28/HDBR31, and HDBR56/HDBR60 for the RSL and COCKTAIL study, respectively. L, left hemisphere; R, right hemisphere.

Supplementary Table 7. Contrasts examining the effect of *Time* on eLORETA resting state functional connectivity for the RSL (n = 23) and COCKTAIL (n = 20) experiment.*

**Experiment**	**Contrast**	$t_{critical}$	t_{max}	p
**RSL**	HDBR2 vs BDC-7	4.21	3.86	0.142
	HDBR28 vs BDC-7	4.23	3.20	0.597
	HDBR56 vs BDC-7	4.19	3.41	0.414
	R+10 vs BDC-7	4.26	2.88	0.872
	HDBR28 vs HDBR2	4.23	3.16	0.668
	HDBR56 vs HDBR2	4.21	3.07	0.756
	HDBR56 vs HDBR28	4.21	2.64	0.989
**COCKTAIL**	HDBR7 vs BDC-8	4.49	3.77	0.281
	HDBR31 vs BDC-8	4.42	3.81	0.224
	HDBR60 vs BDC-8	4.37	3.14	0.757
	R+7 vs BDC-8	4.42	3.16	0.738
	HDBR31 vs HDBR7	4.32	3.95	0.149
	HDBR60vs HDBR7	4.38	3.85	0.199
	HDBR60 vs HDBR31	4.31	3.33	0.533

*Connectivity was defined as the lagged phase synchronization between the intracortical EEG-source estimates of the regions of interest. $t_{critical,}$ critical probability threshold of non-parametric randomization test with 5000 randomizations corrected for multiple comparisons; $t_{max,}$ maximal t-statistic; $p_{corr.}$, p-value.

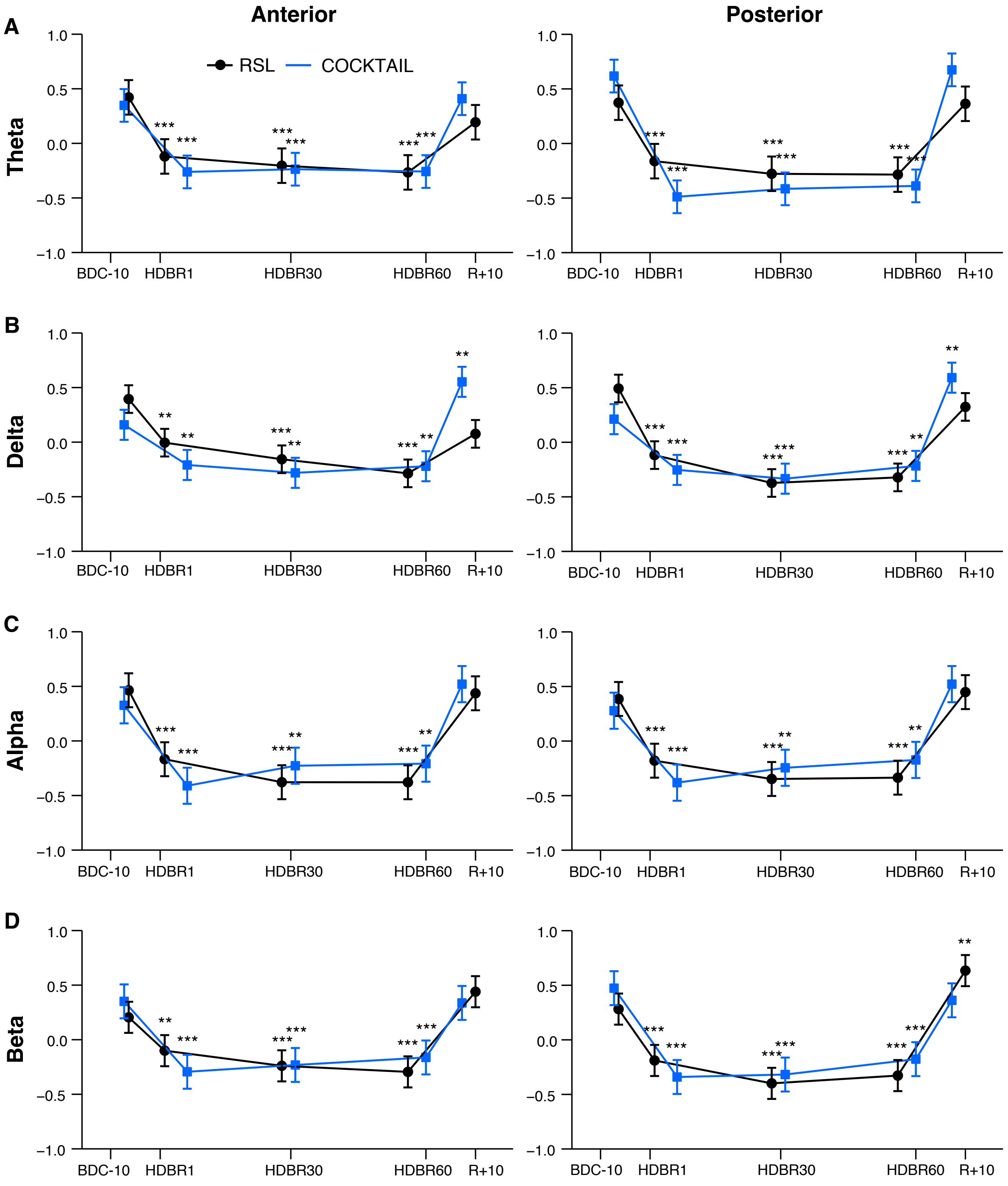

Supplementary Figure 1. Impact of long-duration head-down tilt bed rest on normalized electrocortical activity for the RSL study (*n* = 23, blue circle), and the COCKTAIL study (N = 20, black square). Time courses show changes of EEG spectral power after z-transforming across study participants and testing days for anterior and posterior sites, within the **(A)** theta, **(B)** delta, **(C)** alpha, and **(D)** beta frequency. Data are presented for each time point as estimated marginal means and standard errors. Significant levels with respect to baseline are indicated by asterisks. BDC-10 to BDC-1 refers to baseline data collection. HDBR1, HDBR30, and HDBR60 indicate first, 30th, and 60th day of HDBR. R+0 to R+10 correspond to the first and 11th day after HDBR. For RSL data were collected at BDC-7, HDBR2, HDBR28, HDBR56, and R+10. For COCKTAIL data were collected at BDC-8, HDBR7, HDBR30, HDBR60, and R+7. **p* < 0.05, ***p* < 0.01, and ****p* < 0.001 compared to baseline.
